# A Novel Defined Pyroptosis-Related Gene Signature for Predicting Prognosis and Treatment of Glioma

**DOI:** 10.3389/fonc.2022.717926

**Published:** 2022-03-31

**Authors:** Zhihao Yang, Zhigang Chen, Yu Wang, Zhiwei Wang, Deran Zhang, Xiaoyu Yue, Yinfei Zheng, Lianxin Li, Erbao Bian, Bing Zhao

**Affiliations:** ^1^ Department of Neurosurgery, The Second Affiliated Hospital of Anhui Medical University, Hefei, China; ^2^ Cerebral Vascular Disease Research Center, Anhui Medical University, Hefei, China

**Keywords:** pyroptosis, gene signature, prognosis, treatment, glioma

## Abstract

Pyroptosis, a form of programmed cell death, that plays a significant role in the occurrence and progression of tumors, has been frequently investigated recently. However, the prognostic significance and therapeutic value of pyroptosis in glioma remain undetermined. In this research, we revealed the relationship of pyroptosis-related genes to glioma by analyzing whole transcriptome data from The Cancer Genome Atlas (TCGA) dataset serving as the training set and the Chinese Glioma Genome Atlas (CGGA) dataset serving as the validation set. We identified two subgroups of glioma patients with disparate prognostic and clinical features by performing consensus clustering analysis on nineteen pyroptosis-related genes that were differentially expressed between glioma and normal brain tissues. We further derived a risk signature, using eleven pyroptosis-related genes, that was demonstrated to be an independent prognostic factor for glioma. Furthermore, we used Gene Ontology (GO) and Kyoto Encyclopedia of Genes and Genomes (KEGG) to implement functional analysis of our gene set, and the results were closely related to immune and inflammatory responses in accordance with the characteristics of pyroptosis. Moreover, Gene Set Enrichment Analysis (GSEA) results showed that that the high-risk group exhibited enriched characteristics of malignant tumors in accordance with its poor prognosis. Next, we analyzed different immune cell infiltration between the two risk groups using ssGSEA. Finally, CASP1 was identified as a core gene, so we subsequently selected an inhibitor targeting CASP1 and simulated molecular docking. In addition, the inhibitory effect of belnacasan on glioma was verified at the cellular level. In conclusion, pyroptosis-related genes are of great significance for performing prognostic stratification and developing treatment strategies for glioma.

## Introduction

As the most common malignant primary brain tumor, gliomas comprise approximately 44% of central nervous system tumors (1). Among gliomas, glioblastoma (GBM) is the most aggressive subtype. The median survival of patients diagnosed with GBM is 12–15 months and the 5-years survival rate for GBM patients is less than 5% (2, 3). Despite the existence of a standard therapeutic schedule, including surgery and subsequent radiation and chemotherapy, the prognosis of patients with glioma is still dissatisfactory (4, 5). With the development of molecular biology techniques, our comprehension of glioma pathogenesis has vastly improved, and important changes at the genetic level have been clinically identified. However, outcomes remain unfavorable for glioma patients. Hence, it is of great significance to identify additional molecular markers to accurately evaluate prognosis and to explore more effective ways to treat glioma.

Cell death is a physiological mechanism by which the body maintains its normal function through necrosis and programmed cell death (autophagy and apoptosis) (6). With the discovery of ferroptosis (7), pyroptosis (8), and other types of cell death, the relationship between cell death and disease has attracted renewed attention. Recently, many studies have identified cell death-related biomarkers to predict the prognosis of tumors *via* integrated bioinformatics analysis. For example, a nine autophagy-related gene signature was constructed to evaluate outcomes in oral squamous cell carcinoma patients (9). In kidney renal clear cell carcinoma patients, Wang et al. identified an apoptosis-related gene signature with significant value for predicting OS (10). In addition, for glioma patients, a ferroptosis-related gene signature could potentially predict disease outcomes (11). However, the clinical and biological significance of pyroptosis-related gene signatures has not yet been explored in gliomas.

Pyroptosis is a gasdermin-dependent form of proinflammatory necrotic cell death. Stimulation of caspase-1/4/5/11 (caspase-4/5 are human homologs of murine caspase-11) by different inflammasome pathways leads to their activation by autoprocessing. Active caspases can then cleave gasdermin-D into its N-terminus, GSDMD-N, which translocates to the membrane and induces lytic cell death by forming multisubunit pores (12–16). In addition, activated caspase-1 facilitates the maturation of IL-1β and IL-18, subsequently, leaking through the pores (17). Owing to the lipotropic ability of the N-terminus and its perforation of the cell membrane, other members of the gasdermin family can also induce pyroptosis (18). As a research direction with great promise, pyroptosis plays a crucial role in various diseases including atherosclerosis, neurodegeneration, and tumors (19–21). In tumors, pyroptosis is inflammatory and immunogenic, recruiting and activating multiple immune cells (22, 23). However, inflammation is a double-edged sword that can promote both tumorigenesis and antitumor immunity during all stages of tumor development (24). Cui et al. reported that MST1 inhibits the progression of pancreatic cancer *via* pyroptosis (25). JQ1, a BRD4 inhibitor, suppresses the proliferation and EMT of renal cancer via inducing pyroptosis (26), while a recent study revealed that triggering pyroptosis in the central hypoxic region of cancer provokes the progression of tumors and is associated with reduced survival (27). However, there are few reports concerning glioma and pyroptosis. Therefore, bioinformatics analysis of pyroptosis-related genes may reveal their prognostic value and provide potential therapeutic targets for glioma treatment.

In the present study, we collected pyroptosis-related genes reported in literatures and systematically analyzed their expression levels with respect to different clinicopathological features in gliomas with RNA sequencing data from both TCGA and CGGA datasets. According to the expression profile of pyroptosis-related genes, we identified two subgroups of gliomas with different prognoses and clinical features and established a risk signature that independently predicted the clinical prognosis of glioma patients. Moreover, we examined the relationship between our risk signature and the tumor immune microenvironment. Furthermore, we explored a potential therapeutic target as well as a therapeutic drug for this target.

## Methods

### Patients and Datasets

The TCGA RNA-seq data (670 samples) and clinical follow-up cases (665 samples) were acquired from TCGA database (http://cancergemome.nih.gov/) and served as the training set. Likewise, the quantity of CGGA RNA-seq data was 620, and the corresponding clinical data samples were 619, which were acquired from the CGGA database (http://www.cgga.org.cn) as the validation set. Furthermore, gene expression data of 1140 normal human brain tissue samples were obtained from the GTEx database (http://gtexportal.org/home/).

### Selection of Pyroptosis-Related Genes

We first collected all pyroptosis-related genes from published literature, and then we chose genes that had explicit RNA expression data in both TCGA and GTEx datasets, which generated one hundred and forty-four pyroptosis-related genes (**Table 1**). Next, we identified differentially expressed pyroptosis-related genes between the two datasets using the R package “BiocManager”.

**Table 1:** One hundred and forty-four pyroptosis-related genes collected from published literature.

TIGAR	NOD2	NEDD4	BAX	ZBP1	BRD4	RIPK3	TP53
**PRKACB**	ADCY4	DPP8	SOD1	MAPK8	CLEC4E	TNFRSF4	MAP3K7
**PDCD4**	TICAM1	GSDMA	PTGER2	DDX3X	NLRC5	BRCC3	CTSB
**RHEB**	CARD8	NLRP9	POP1	ADRA2B	HDAC6	HSPA12A	HIF1A
**TYK2**	TLR2	NFKB2	NLRC4	BNIP3	HK1	HMOX1	NEK7
**STAT1**	FOXO1	GZMA	PRKACA	RELA	FADD	KCNK6	IRGM
**CASP4**	CYCS	ATG7	GSDMD	RIPK1	LDLR	NLRP2	AOAH
**NLRP1**	DAPK3	CASP9	NFKB1	MAPK1	IRF1	AIM2	SERPINB1
**RELB**	TRAF6	PDCD6IP	PRKAA1	GBP3	CASP6	PYDC2	NAIP
**P2RX4**	IRF2	P2RX7	IL18	PELI2	GSDMC	SDHB	GSDMB
**SENP6**	EP300	PCSK9	IL1β	NLRP3	TNF	TOMM20	IRAK1
**IFI16**	SENP7	MUL1	MEFV	NFE2L2	CD63	TXNIP	DHX9
**IRF8**	PRKACG	TLR3	KLF2	IL13	GPX4	MALT1	CAMP
**GBP2**	NOS2	NLRP12	IRF3	JOSD2	NLRX1	PYCARD	PANX1
**SARM1**	CASP3	NLRP7	JAK1	CASP5	TLR4	REL	RHOA
**IRAK4**	NR1H2	GBP4	KAT2B	MAPK14	PDE8A	CD274	TNFRSF1A
**DDX58**	HMGB1	GBP1	SYK	BTK	CASP1	GZMB	CDC37
**IFNG**	CASP8	NLRP6	PTGER4	CYLD	DPP9	STAT3	MYD88

### Consensus Clustering Analysis

The R package “ConsensusClusterPlus” was used to conduct the consensus clustering to cluster the glioma patients into different groups. The cumulative distribution function (CDF) and consensus matrices were implemented to evaluate the optimum number of subgroups (28).

### Construction of Risk Prognosis Signature

To explore the prognostic value of pyroptosis-related genes, we first implemented univariate Cox regression analysis of nineteen differentially expressed pyroptosis-related genes and identified fourteen genes significantly related to survival. Then, the LASSO Cox regression algorithm (29) was utilized to build a risk gene signature. Finally, eleven pyroptosis-related genes and their corresponding coefficients were identified, and the penalty parameter (λ) associated with the smallest 10-fold cross validation within the training set was decided using the minimum criteria. The risk score of every glioma patient was calculated by utilizing the following formula:


$$\text{Risk score}=\Sigma_{\text{x}=1}^{\text{n}}\text{ Coef x}*\text{Yx}$$

Coef_x_ is the regression coefficient belonging to gene X, and Y_X_ is the expression level of gene X. In light of the risk score, the median risk score for glioma patients in the two datasets could be identified respectively. Then, patients with a risk score above the median value were classified as the high-risk group, while the remaining glioma patients were identified as the low-risk group.

### Functional Enrichment Analysis

GO and KEGG enrichment analyses were based on risk scores and used to perform the functional enrichment analysis of our risk model. 902 related genes were then selected using the Pearson correlation of R software by setting |correlation coefficient| > 0.68 and P < 0.001 as cut-offs and further obtained biological functions associated with the risk signature defined by pyroptosis-related genes through the R package “clusterprofler”. GSEA was conducted using GSEA software (http://software.broadinstitute.org/gsea/index.jsp) to explore the functions most likely to be affected by the eleven pyroptosis-related genes.

### Tumor Purity Estimation and Immune Infiltration

We evaluated tumor purity using the R package “ESTIMATE,” which is based on the estimation of stromal and immune cell markers (30). ssGSEA was performed to explore the different infiltration degrees of 24 immune cell types in the two risk groups using the R package “GSVA”.

### Constructing the Protein-Protein Interaction (PPI) Network and Identifying Potential Drugs

The STRING database contains a great deal of protein interaction information and was utilized to build the PPI network (https://string-db.org/). We entered the names of eleven pyroptosis-related genes and selected Homo sapiens as the species. Then, we set the minimum required interaction score to 0.4 (default) as the screening condition to construct the PPI network. The sources of active protein interactions included coexpressions, experiments, cooccurrence, text mining, neighborhoods, gene fusions and databases. The Drug Gene Interaction Database (DGIdb) (31) is an available resource that includes the drug targeted genome and drug–gene interactions (https://www.dgidb.org).

### Molecular Docking of Core Target and Compound to Simulate the Binding

The PDB files of the core target were downloaded from the RCSB PDB website (http://www.rcsb.org) and 3D structures of the belnacasan ligand were downloaded from the PubChem website (https://pubchem.ncbi.nlm.nih.gov). Then, we conducted molecular docking by utilizing Dockthor (32), a web tool for ligand–protein docking. The docking algorithm was set as the local search parameter employing Python 2.5, and PyMOL was employed to visualize the results.

### Cell Culture and Reagents

Glioma cell lines, including LN-18 and T98G and normal astrocyte HEB, were all purchased from the Chinese Academy of Sciences. Glioma and HEB cells were cultured in DMEM (Gibco, USA) containing 10% fetal bovine serum (FBS; Gibco, USA) and placed in an incubator at a constant temperature of 37°C and carbon dioxide concentration of 5%. The medium was changed every 48-72 hours. Cell passaging was performed when cell density reached 80% of the cell culture flask. The CCK-8 reagent was purchased from Abcam Biotechnology, and belnacasan was purchased from GLPBIO company.

### Cell Counting Kit 8 (CCK-8) Assay

To evaluate the effect of belnacasan on cell viability, 100 μl of suspended cells were added to 96-well plates (1000 cells/well). Cells were incubated in 96-well plates treated with the indicated concentrations (0 μM, 5 μM, 10 μM, 20 μM and 40 μM) of belnacasan for 48 hours. Then, 10 μL of the CCK-8 working solution was added to the corresponding wells. After incubating under the aforementioned cell culture conditions for 2 hours, the absorbance was measured using a microplate reader at 460 nm. Finally, employed a formula to evaluate cell viability.

### Cell Migration and Invasion Assay

For the cell migration assay, 1×10^4^ cells were seeded in a Transwell chamber without Matrigel and placed in a 24-well plate, and 600 μl serum-supplemented medium was added to the corresponding chamber. For the cell invasion assay, 8×10^4^ cells were seeded in a Transwell chamber with Matrigel and placed in the chamber of a 24-well plate with 600 μl serum-supplemented medium. Cells were all suspended in serum-free medium. After incubating for 8 hours for migration assay and 48 hours for the invasion assay in an incubator at 37°C, nonmigrating or noninvasive cells were removed from the parietal chamber using a cotton swab. Then, 4% paraformaldehyde was used to immobilize migrated and invaded cells for approximately 25 minutes. After drying, the cells were stained with crystal violet at a concentration of 0.5% for 15 minutes. Finally, inverted phase contrast microscope was used to calculate the quantity of cells that passed through the membrane of the Transwell chamber (Olympus, Japan).

### Statistical Analysis

All experimental data are expressed as the mean ± SD conducted at least three times. Student’s t-test or one‐way ANOVA was used for comparisons between groups of continuous variables. Kaplan-Meier analysis was used to analyze the OS differences between the two groups by conducting the log-rank test. Differences in clinicopathological characteristics were detected by performing the chi-square test. Independent prognostic factors of the risk score were judged by performing univariate and multivariate analyses. ROC analysis using the R package “survivalROC” was performed to judge whether the risk model could accurately predict survival. GraphPad Prism or R software was used to perform statistical analyses. *P* < 0.05 indicates statistical significance.

## Results

### Identification of Pyroptosis-Related Genes

**Figure 1:**
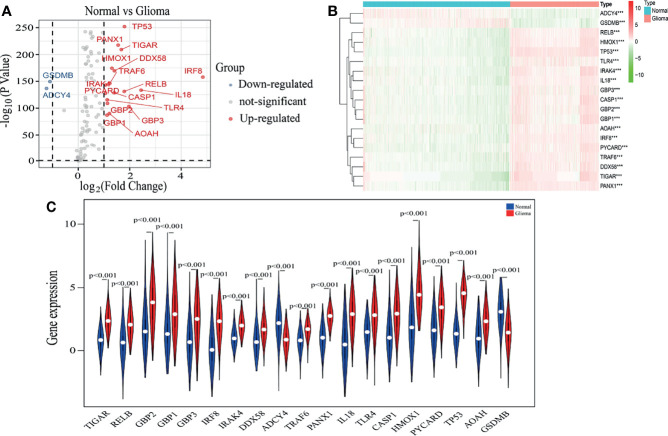
Identification of pyroptosis-related genes. **(A)** A volcano plot of 144 differentially expressed pyroptosis-related genes, including nineteen selected genes. **(B)** The heatmap shows expression levels of nineteen pyroptosis-related genes in normal brain and glioma tissues. ***p < 0.001. **(C)** The vioplot shows differential expression of nineteen pyroptosis-related genes between normal brain and glioma tissues.

As described in the MATERIALS AND METHODS, we identified nineteen pyroptosis-related genes using |log_2_FC|>1 and P<0.01 as cut-offs (**Figure 1A**). The heatmap in **Figure 1B** displays the expression levels of the nineteen pyroptosis-related genes in normal brain and glioma tissues. We further observed differential expression of the nineteen pyroptosis-related genes using vioplot, of which seventeen pyroptosis-related genes were more highly expressed in glioma tissues, including IRAK4, RELB, HMOX1, TP53, TLR4, IL18, GBP1, GBP2, GBP3, CASP1, AOAH, IRF8, PYCARD, TRAF6, DDX58, TIGAR and PANX1, while expression of GSMB and ADCY4 was higher in normal tissues (**Figure 1C**).

### Consensus Clustering of Pyroptosis-Related Genes Identified Two Clusters of Glioma Patients With Different Clinicopathological Features and Clinical Prognoses

**Figure 2:**
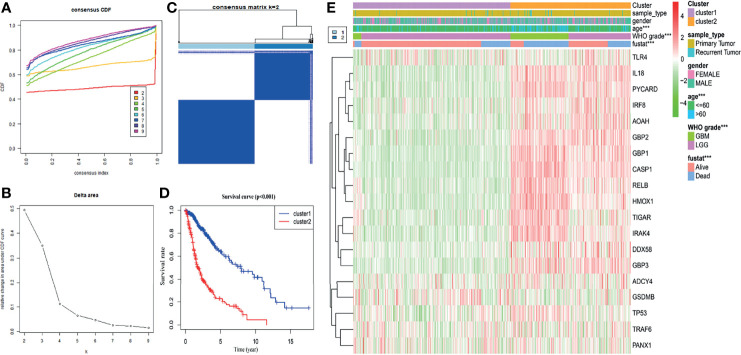
Glioma classification with differential OS and clinicopathological features in the TCGA dataset. **(A)** Cumulative distribution function of consensus clustering for k = 2 to 9. **(B)** The variation in area under the CDF curve for k = 2 to 9. **(C)** The consensus clustering matrix showed that the 670 glioma patients from the TCGA dataset were classified into two clusters (k = 2). **(D)** The Kaplan–Meier OS curves of cluster 1/2. **(E)** Differential clinicopathologic features and nineteen pyroptosis-related gene expression levels between cluster 1 and cluster 2. ***p < 0.001.

Based on the expression similarity in nineteen pyroptosis-related genes, k = 2 was an adequate selection with stable clustering in the TCGA dataset (**Figures 2A–C**). In addition, the cluster 2 subgroup exhibited a markedly shorter OS than the cluster 1 subgroup (**Figure 2D**). Next, we compared the clinicopathological features of cluster 1 and cluster 2. The cluster 1 subgroup primarily exhibited younger age at diagnosis, lower grade tumors and alive status while the cluster 2 subgroup was significantly correlated with older age, GBM phenotype and dead status (**Figure 2E**). Meanwhile, different clusters distributed glioma patients with the same grade, indicating that gliomas of the same grade exist heterogeneity.

Next, in the CGGA database, we also performed cluster analysis based on the nineteen genes selected by TCGA database. Our results showed that classifying the cases into two clusters was appropriate (**Supplementary Figures 1A-C**). In addition, OS in the cluster 2 subgroup was shorter than in the cluster 1 subgroup (**Supplementary Figure 1D**). Combined with clinical outcomes and clinicopathological features, the cluster 1 subgroup was significantly correlated with lower grade and alive status. In contrast, the cluster 2 subgroup primarily contained gliomas with a GBM phenotype and death status (**Supplementary Figure 1E**). These results are all consistent with those observed in the TCGA database.

### Construction of a Risk Signature Containing Eleven Selected Pyroptosis-Related Genes

**Figure 3:**
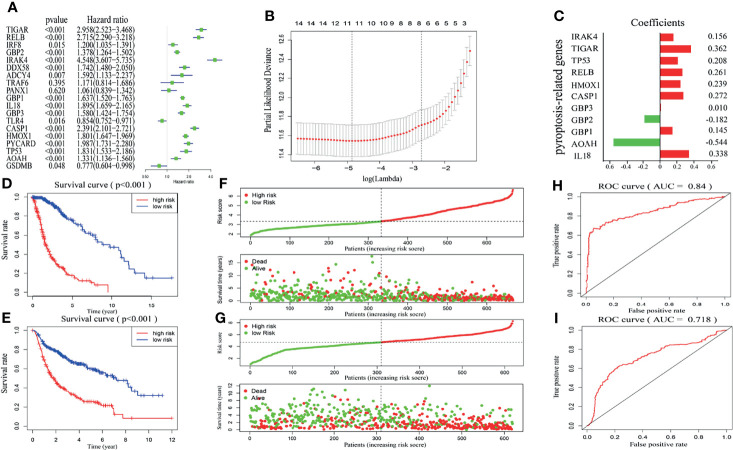
Construction and validation of risk model. **(A)** Univariate analysis presents the hazard ratios and P-value of nineteen pyroptosis-related genes by the forest plot. **(B)** Cross-validation for adjusting parameter choice in LASSO regression analysis. **(C)** The risk signature containing eleven pyroptosis-related genes and the corresponding coefficients. **(D, E)** Kaplan–Meier survival curves for glioma patients from the TCGA **(D)** and CGGA **(E)** datasets in high- and low-risk groups. **(F, G)** Surveying the risk model in TCGA **(F)** and CGGA **(G)** datasets: distribution and survival status in glioma patients between the two risk groups. **(H, I)** The predictive efficiency of the risk score is presented by the ROC curves in both TCGA **(H)** and CGGA **(I)** datasets.

We next sought to explore the prognostic effect of pyroptosis-related genes in gliomas. First, univariate analysis was used to preliminarily screen genes that were identified as being related to survival based on expression levels of nineteen genes in the TCGA dataset (**Figure 3A**). The results revealed that fourteen out of nineteen genes were obviously associated with OS and met the criteria of P < 0.01. Next, by conducting LASSO Cox regression analysis of fourteen pyroptosis-associated genes in the TCGA dataset regarded as the training set, eleven pyroptosis-related genes were selected to construct a risk model based on the minimum criteria to better predict the clinical prognosis of glioma patients (**Figure 3B**). Moreover, the corresponding coefficients acquired from the LASSO analysis were employed to calculate the risk score of each glioma patient in the TCGA and CGGA datasets. The eleven genes were IL18, AOAH, GBP1, GBP2, GBP3, CASP1, HMOX1, RELB, TP53, TIGAR and IRAK4 with coefficients of 0.337528261, -0.543539888, 0.144892417, -0.182129339, 0.010353371, 0.272493354, 0.238556334, 0.261383001, 0.208246149, 0.362501856 and 0.155829151, respectively (**Figure 3C**).

In light of the median risk score, glioma patients in the training and validation sets were respectively separated into low- and high-risk groups. Then, differences in OS were compared between the two categories, and we found that OS in the low-risk group was markedly higher than in the high-risk group in both the TCGA and CGGA datasets (**Figures 3D, E**). **Figures 3F, G** further shows the glioma patient distribution of our risk model in the TCGA and CGGA datasets. Furthermore, the ROC curve showed that our risk signature was very accurate and meaningful, and the AUCs of TCGA and CGGA were 84% and 71.8%, respectively (**Figures 3H, I**). These results indicate that our risk signature constructed by pyroptosis-related genes accurately predicts disease outcomes in glioma patients.

### The Clinical Information and Prognostic Impact of a Pyroptosis-Related Gene Signature in Glioma

**Figure 4:**
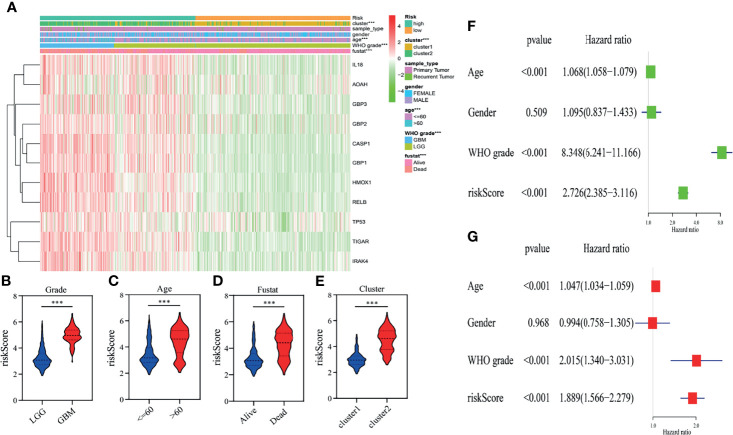
Relationship between the clinicopathological characteristics, cluster 1/2 groups and risk score in the TCGA dataset. **(A)** The heatmap displays the differential distribution of clinicopathological features and eleven pyroptosis-related gene expression levels in gliomas with low and high risk. **(B–E)** The WHO grade **(B)**, age **(C)**, fustat **(D)**, and cluster 1/2 subgroups **(E)** stratify the TCGA dataset, and the distribution of risk scores is shown. ***p < 0.001. **(F, G)** Univariate **(F)** and multivariate **(G)** analyses for the TCGA cohort including age, gender, WHO grade and risk score.

To demonstrate the relationship between the gene signature established by eleven pyroptosis-related genes and clinical information, patients were classified based on their risk score, and marked differences in the distribution of the grade, age, fustat and cluster were observed. The high-risk group primarily contained glioma patients with older age, GBM phenotype, death status and cluster 2 in both TCGA (**Figure 4A**) and CGGA (**Supplementary Figure 2A**) datasets (**Table 2**). Furthermore, the eleven pyroptosis-related genes were all highly expressed in the high-risk group in both datasets. We next compared the values of risk scores belonging to glioma patients divided by clinical characteristics. Results indicated that the values of risk scores were quite diverse in glioma patients separated based on WHO grade, age, fustat, and cluster 1/2 subgroups in TCGA (**Figures 4B–E**) and CGGA (**Supplementary Figures S2B–E**) datasets. In addition, high risk scores were related to IDH wild type genotype, 1p19q noncodel, nonchemotherapeutic status and recurrent glioma in the CGGA dataset (**Supplementary Figures 2F–I**).

**Table 2:** Correlation between risk scores and clinicopathological factors of glioma patients in the two cohorts.

Training set (TCGA database, n = 665)			
Features	Low-risk group	High-risk group	P-value
	n = 333	n = 332	
**Gender**			
** Male**	178	189	ns
** Female**	155	143	
**Age**			
** ≤ 60**	305	221	<0.001
** > 60**	28	111	
**Grade**			
** LGG**	332	174	<0.001
** GBM**	1	158	
**Survival state**			
** Alive**	277	140	<0.001
** Dead**	56	192	
**Sample Type**			
** Primary tumor**	320	318	ns
** Recurrent tumor**	13	14	
**Cluster**			
** Cluster 1**	300	76	<0.001
** Cluster 2**	33	256	
**Validation set (CCGA database, n = 619)**			
**Features**	**Low-risk group**	**High-risk group**	**P-value**
	**n = 310**	**n = 309**	
Gender			
Male	173	183	ns
Female	137	126	
Age			
≤ 60	289	269	<0.05
> 60	21	40	
Grade			
II	110	63	<0.001
III	141	90	
IV	59	156	
Survival state			
Alive	197	99	<0.001
Dead	113	210	
Sample Type			
Primary tumor	223	178	<0.001
Recurrent tumor	87	131	
Cluster			
Cluster 1	255	44	<0.001
Cluster 2	55	265	

P < 0.05 indicates a statistically significant difference; ns indicates no significance.

We then performed univariate and multivariate analyses in the TCGA training dataset to determine whether our risk model represented an independent prognostic factor. As shown in the results, the risk score, age and grade were all associated with OS (**Figures 4F, G**). The same results were observed in the CGGA validation dataset. According to the results of univariate and multivariate analyses, the risk score, age and WHO grade were all obviously associated with OS (**Supplementary Figures 2J, K**). Taken together, these results validated that our risk model derived from eleven pyroptosis-related genes independently predicts prognosis in glioma patients.

### Functional Enrichment Analysis of the Risk Signature

**Figure 5:**
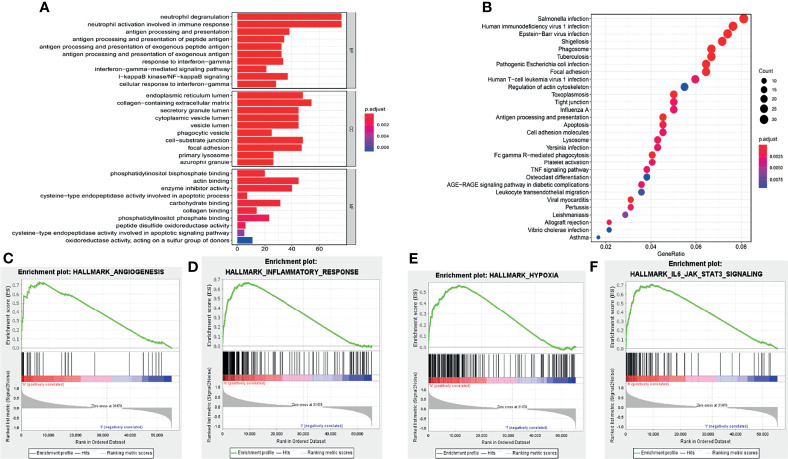
Functional analysis of the risk signature. **(A)** GO analysis of eleven pyroptosis-related genes. “BP”, “CC” and “MF” represent “biological process”, “cellular component” and “molecular function”, respectively. **(B)** KEGG analysis of eleven pyroptosis-related genes. **(C–F)** GSEA showed that genes with higher expression in the high-risk group enriched the features of malignant tumors.

GO and KEGG analyses were performed for the eleven pyroptosis-related genes to explore potential biological processes associated with glioma. GO analysis showed that the eleven pyroptosis-related genes were primarily associated with neutrophil activation involved in the immune response, neutrophil degranulation, antigen processing and presentation and IkappaB kinase/NF−kappaB signaling (**Figure 5A**). The results of KEGG analysis revealed that these genes were primarily related to infection, phagosomes, focal adhesion and antigen processing and presentation (**Figure 5B**). Moreover, GSEA was applied to compare the high- and low-risk groups. We discovered that the high-risk group was closely related to angiogenesis, inflammation, hypoxia and IL6/JAK/STAT3 signaling compared to the low-risk group (**Figures 5C–F**). These results all indicate that the two risk groups identified by eleven pyroptosis-related genes were correlated with the characteristics of pyroptosis and glioma malignancy.

### Tumor Purity and Immune Microenvironment of the Risk Signature

**Figure 6:**
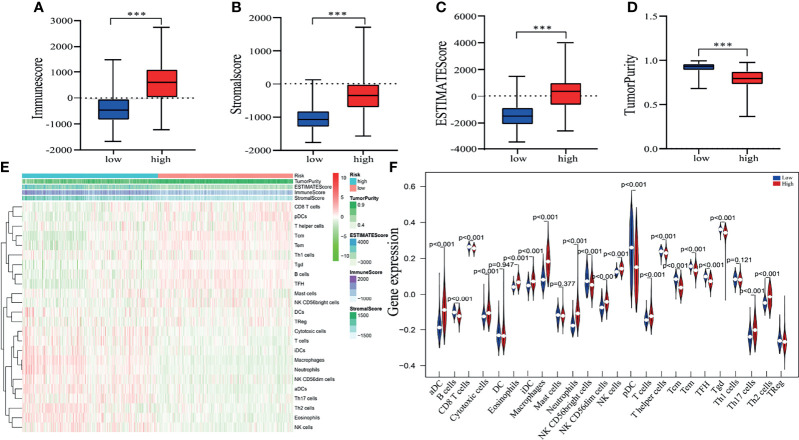
Estimating tumor purity and TIIC (tumor-infiltrating immune cell) components. **(A–D)** There was a significant difference in the immune score **(A)**, stromal score **(B)**. ESTIMATE score **(C)** and tumor purity **(D)** between high- and low-risk patient groups. ***p < 0.001. **(E)** The heatmap shows the tumor purity, corresponding immune cell infiltrates, immune score, ESTIMATE score and stromal score for each glioma patient in the two risk groups. **(F)** The vioplot revealed the different proportion of each immune cell between the two risk groups (blue was the low-risk group, and red was the high-risk group).

Pyroptosis is closely associated with immunity, thus, the expression profile of glioma was analyzed by adopting the ESTIMATE algorithm. Then, we calculated the immune score, ESTIMATE score, stromal score, and tumor purity for each glioma patient. The box chart shows that the low-risk group had significantly higher tumor purity and lower immune scores, ESTIMATE scores and stromal scores than the high-risk group (**Figures 6A–D**). We further explored the relationship of immune cell infiltration between the two risk groups using the ssGSEA method. Our results indicated that activated dendritic cells (aDCs), cytotoxic cells, eosinophils, immature dendritic cells (iDCs), macrophages, neutrophils, NK CD56dim cells, NK cells, T cells, Th17 cells, and Th2 cells (all p < 0.001) exhibited higher proportions in the high-risk group than in the low-risk group (**Figures 6E, F**). The relative proportions of CD8 T cells, NK CD56bright cells, plasmacytoid dendritic cells (pDCs), T helper cells, T gamma delta (Tgd), T central memory (Tcm), T effector memory (Tem), T cell follicular helper (Tfh), B cells (all p < 0.001), and T cell regulatory (Treg) (p = 0.019) were significantly upregulated in the low-risk group (**Figures 6E, F**).

### Eleven Pyroptosis-Related Genes Detection and Validation

**Figure 7:**
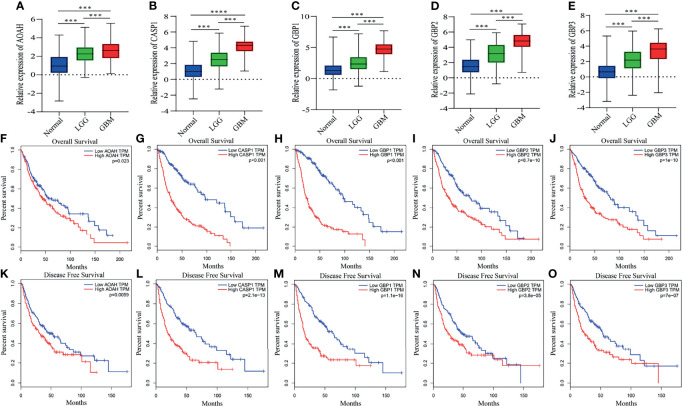
Differential expression, OS and DFS of five pyroptosis-related genes. **(A–E)** Boxplots showing the relative expression levels of AOAH **(A)**, CASP1 **(B)**, GBP1 **(C)**, GBP2 **(D)** and GBP3 **(E)** in normal brain tissues, low-grade glioma tissues and GBM tissues. ***p < 0.001 and ****p < 0.0001. **(F–O)** Survival curves for OS and DFS in glioma patients with high expression and low expression of the following genes: AOAH **(F, K)**, CASP1 **(G, L)**, GBP1 **(H, M)**, GBP2 **(I, N)** and GBP3 **(J, O)**.

The eleven pyroptosis-related genes were next validated using glioma data from the GEPIA database. Among them, expression levels of all eleven genes were markedly higher in GBM or low-grade glioma tissues than in normal brain tissues. Moreover, in accordance with the GEPIA database, expression levels of these eleven genes were negatively correlated with OS and disease-free survival (DFS) in glioma patients (**Figure 7** and **Supplementary Figure 3**). These results are consistent with our previous results.

### Identification of the Core Gene and Molecular Docking Simulation

**Figure 8:**
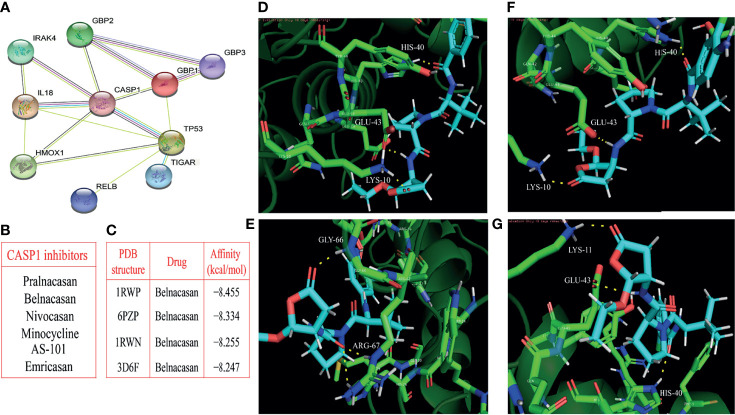
Identification of the core gene and simulated molecular docking. **(A)** Construction of the CASP1-centered protein-protein interaction network. **(B)** Six inhibitors targeting CASP1. **(C)** Four docking with the strongest affinities. The molecular docking of belnacasan ligand and four PDB structures of CASP1 including 1RWN, 1RWP, 3D6F and 6PZP, the amino acid residue generating hydrogen bonds with belnacasan are HIS-40, GLU-43 and LYS-10 for 1RWN **(D)**, GLY-66 and ARG-67 for 1RWP **(E)**, HIS-40, GLU-43 and LYS-10 for 3D6F **(F)**, LYS-11, GLU-43 and HIS-40 for 6PZP **(G)**. The molecule in blue is belnacasan, the bright green is the amino acid residue of CASP1 docking with belnacasan, and the yellow dotted lines represent the hydrogen bond.

The PPI network contained nodes and edges, as shown in **Figure 8A**. The nodes represent the eleven pyroptosis-related genes and the edges represent the interactions between the genes. A higher degree of CASP1 represented the core gene in the PPI network and might be more closely related to glioma prognosis. Next, we sought to identify drugs that can pharmacologically target this core gene and explored whether CASP1 can serve as a therapeutic target of glioma using specific drugs. We searched DGIdb and found six inhibitors targeting CASP1 (**Figure 8B**). Belnacasan, the most widely used and available CASP1 inhibitor, was chosen to perform the molecular docking simulation.

We obtained the PDB files of CASP1 and the 3D conformer of the belnacasan ligand from the corresponding website. CASP1 resulted in a total of thirty available PDB structures for docking, and Dockthor generated a series of docking modes and affinities, as shown in **Table 3**. Based on the docking results, the four strongest affinities were −8.455 kcal/mol, −8.334 kcal/mol, −8.255 kcal/mol and −8.247 kcal/mol, which belong to 1RWP, 6PZP, 1RWN and 3D6F docking, respectively, with belnacasan (**Figure 8C**). Visualization of the docking results is shown in **Figures 8D–G**.

**Table 3:** Interaction force of belnacasan with all PDB structures of the CASP1 gene.

PDB structure	Drug	Affinity (kcal/mol)	PDB structure	Drug	Affinity (kcal/mol)
1BMP	Belnacasan	-7.431	2H54	Belnacasan	-7.910
1RWK	Belnacasan	-7.741	2HBQ	Belnacasan	-7.528
IRWM	Belnacasan	-7.204	2HBR	Belnacasan	-7.945
1RWN	Belnacasan	-8.255	2HBY	Belnacasan	-8.180
1RWO	Belnacasan	-8.043	2HBZ	Belnacasan	-7.749
1RWP	Belnacasan	-8.455	3D6F	Belnacasan	-8.247
1RWV	Belnacasan	-7.636	3D6H	Belnacasan	-8.090
1RWW	Belnacasan	-7.668	3D6M	Belnacasan	-7.830
1RWX	Belnacasan	-8.108	3NS7	Belnacasan	-7.512
1SC3	Belnacasan	-8.114	5MMV	Belnacasan	-8.096
2FQQ	Belnacasan	-7.019	5MTK	Belnacasan	-7.849
2H4W	Belnacasan	-8.229	6BZ9	Belnacasan	-7.740
2H4Y	Belnacasan	-7.859	6F6R	Belnacasan	-7.482
2H48	Belnacasan	-8.203	6KN0	Belnacasan	-6.729
2H51	Belnacasan	-8.049	6PZP	Belnacasan	-8.334

### Belnacasan Significantly Suppresses Glioma Cell Viability, Migration and Invasion

**Figure 9:**
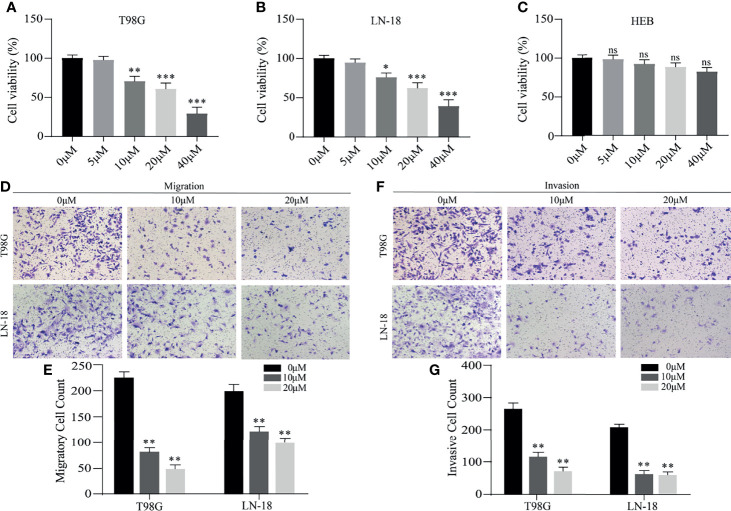
The effects of belnacasan on cell viability, migration and invasion. **(A–C)** T98G, LN-18 and HEB cells were treated with different concentrations of belnacasan for 48 hours, and then CCK-8 assays were performed to determine the cell viability. *p < 0.05, **p < 0.01, and ***p < 0.001 vs 0 μM; ns indicates no significance. **(D, E)** Migratory T98G and LN-18 glioma cells treated with belnacasan at proper concentrations were tested. **p < 0.01 vs 0 μM. **(F, G)** Invasive T98G and LN-18 glioma cells treated with belnacasan at proper concentrations were tested. Scale bar, 200μm. **p < 0.01 vs 0 μM.

We further explored the potential therapeutic effects of belnacasan on glioma. First, the human glioma cell lines T98G and LN-18 were treated with a series of concentrations of belnacasan for 48 hours, and then cell viability was detected by CCK-8. As shown in **Figures 9A, B**, in the presence of belnacasan, cell viability was decreased in a concentration-dependent manner in T98G and LN-18 glioma cells. Moreover, belnacasan demonstrated no toxic effects on the viability of normal brain cells (**Figure 9C**). Given the obvious inhibitory effect on cell viability of belnacasan at concentrations of 10 μM and 20 μM, which maintained cell viability of greater than 60% at these two concentrations, 10 μM and 20 μM were selected for subsequent experiments in both T98G and LN-18 glioma cells. Finally, we conducted cell migration and invasion assays. As shown in **Figures 9D–G**, glioma cells’ ability to migrate and invade was significantly reduced after treatment with belnacasan at different concentrations. These results indicate that belnacasan effectively inhibits the proliferation, migration and invasion of glioma cells.

## Discussion

Glioma is the most pernicious type of primary brain tumor due to malignant progression and a high recurrence rate (33–35). Despite treatment progress with surgery, radiation and chemotherapy, the therapeutic effect of glioma remains unsatisfactory (36). Recently, studies on the molecular characteristics of glioma have proposed many potential markers that can be used to classify glioma, judge prognosis and guide treatment (37), but they are insufficient to predict convoluted glioma prognosis alone and lack the ability to identify effective treatment.

Cell death plays a central role in all aspects of life and is involved in the development of multicellular organisms and tissue homeostasis. Moreover, it is associated with multiple diseases that are caused by deregulated or dysfunctional cell death signaling, including tumors (38). Consequently, there is a growing interest in the relationship between cell death and tumors. Guo et al. built an autophagy-related five-gene signature that has value for judging prognosis in lower-grade glioma patients (37). In addition, ferroptosis acts as a new marker for diagnosis and prognostic judgment in low-grade glioma (39). As one of the most well characterized cell death pathways, the principal characteristics of pyroptosis include cell swelling, membrane perforation, release of cell contents, chromatin condensation and DNA fragmentation (40, 41). Pyroptosis exerts dual effects on tumor. On the one hand, releasing inflammatory cytokines and activated pathways associated with inducing pyroptosis facilitate tumor growth, invasion and drug resistance (42, 43). On the other hand, inducing pyroptosis can directly suppress the tumor proliferation (44). However, to date, molecular subtyping and prognostic models based on pyroptosis-related genes have not been reported in glioma.

A previously reported immune-related gene signature was successfully established to evaluate survival rates in glioma patients (45). Xu et al. constructed an autophagy-related gene signature that acts as an independent prognostic biomarker in glioma (46). Herein, two datasets (TCGA and CGGA) were used to determine the prognostic value of pyroptosis-related genes in glioma patients. First, we screened nineteen genes by collecting previously reported pyroptosis-related genes and performed differential expression analysis between normal and tumor tissues. Based on the expression of nineteen pyroptosis-related genes, we identified two glioma subgroups, cluster 1and cluster 2, by applying consensus clustering analysis. The cluster 1/2 subgroups exhibited different prognoses and clinicopathological features. In addition, Zhou et al. used the expression profiles of immune-related genes to identify three subgroups of diffuse glioma, which were demonstrated to be valid and preferable prognostic factors (47). Next, we performed univariate Cox analysis to screen OS-related genes and utilized the Lasso regression model to subsequently obtain the regression coefficients. Whereafter, a prognostic risk signature with eleven selected pyroptosis-related genes was derived, which stratified the glioma patients into high- and low-risk groups by comparing the risk score of each patient to the median value. Furthermore, univariate and multivariate analyses revealed that the risk score derived from eleven pyroptosis-related genes accurately estimated the prognosis of glioma. Moreover, compared to a single-gene predictive biomarker, the integration of multiple gene markers into a single model enhances the predictive accuracy. However, the specific roles of these eleven pyroptosis-related genes in the pyroptosis pathway with respect to glioma remain uncertain and deserve further study.

In view of the important role of our risk signature in predicting the prognosis of gliomas, we further explored the potential mechanisms. The results of functional analysis revealed that the biological processes of immune and inflammatory responses were abundant in the high-risk group, suggesting an interaction between the pyroptosis-related gene signature and the glioma immune response. This is consistent with pyroptosis being both inflammatory and immunogenic (22, 23). Abundant glioma-associated nontumor cells play important roles in the development of glioma and are represented by stromal and immune cells within the microenvironment of glioma tissues (48, 49). Studies have revealed that stromal cells are closely associated with glioma proliferation, invasion, and angiogenesis (50–53), and increasing evidence indicates that infiltrating immune cells play diverse roles in glioma (54, 55). Moreover, a previous study revealed that low tumor purity is related to unfavorable prognosis in glioma (56). Our ESTIMATE algorithm revealed that the high-risk group exhibited reduced tumor purity and increased stromal and immune scores. Next, we compared the abundance of 24 types of tumor-infiltrating immune cells (TIICs) in the high and low-risk groups. Enrichment of CD8 T cells and NK cells, representing antitumor cells, obviously extends patient survival (57, 58). In addition, enrichment of macrophages generally contributes to the growth, invasion and grade progression of glioma (59). Neutrophils, which inhibit the cytolytic activity of NK cells and CD8 T cells, are also positively correlated with increasing histopathologic grade, reduced survival and treatment resistance (60–62). Consistent with these conclusions, our results showed that the high-risk group had a lower abundance of CD8 T cells, while the low-risk group had a lower abundance of macrophages and neutrophils. However, our high-risk group exhibited higher enrichment of NK cells, and we suspect that this discrepancy might be due to the essential role of NK cells in the tumor microenvironment, where they regulate the overactivated inflammatory response induced by pyroptosis.

In our risk model, expression levels of eleven selected genes were all negatively correlated with favorable outcomes. Moreover, functional analysis revealed that the defined pyroptosis-related genes contributed to cancer progression, which provides strong evidence for molecular targeted treatment of glioma. We further identified the core gene CASP1. CASPs are evolutionarily ancient intracellular proteases that are prevalent in multicellular organisms (63). However, the function of CASP family members in the occurrence and progression of tumors has not been confirmed. According to a previous report, CASP1 is related to comparatively lower survival of pancreatic cancer patients (64). CASP1, one of inflammatory caspases, triggers pyroptosis (65). However, in the absence of GSDMD, CASP1 initiates apoptosis (66). In glioma, the study of Jiang et al. has confirmed that CASP1 mediated pyroptosis (67). Belnacasan, known as a caspase-1 inhibitor, can effectively suppress its activity. Molecular docking simulation revealed that the PDB structure of CASP1 docked well with belnacasan. We further verified the inhibitory effect of belnacasan on glioma cells by CCK-8 and migration and invasion experiments, but the viability of normal astrocytes was not affected by administration of belnacasan. In view of the fact that pyroptosis is inflammatory, and the inflammatory factors released by pyroptosis can promote tumor growth and invasion (42, 43), we speculate that belnacasan reduces the release of inflammatory factors by inhibiting pyroptosis, thereby inhibiting the proliferation and invasion of glioma cells. However, the exact mechanism needs to be further explored. In addition, animal experiments are necessary in future studies to verify the inhibitory effect of belnacasan on glioma *in vivo*. In general, our results revealed that the CASP1 gene may represent a potential therapeutic target and that belnacasan might be a potential therapeutic drug for glioma.

In summary, we identified two subgroups of glioma patients with disparate prognostic and clinical features based on nineteen pyroptosis-related gene expression profiles and developed an eleven pyroptosis-related gene expression-based risk signature with a powerful ability to predict glioma prognosis. Furthermore, we identified a potential therapeutic target and a drug that binds that target. In short, our research adds guidance value to the analysis of glioma prognosis and clinical treatment.

## Data Availability Statement

The original contributions presented in the study are included in the article/**Supplementary Material**. Further inquiries can be directed to the corresponding authors.

## Author Contributions

BZ, EB, and ZY designed this article. ZC conducted experience. YW, ZW, and DZ analyzed the data. ZY, XY, and YZ drafted the manuscript. LL was responsible for article figures. EB revised the manuscript. All authors contributed to the article and approved the submitted version.

## Funding

This research was supported by the National Natural Science Foundation of China (No. 81972348), Key Research and Development Plan Project of Anhui Province (No. 1804h08020270), College Excellent Youth Talent Support Program in Anhui Province (No. gxypZD2019019), Key Projects of Natural Science Research in Anhui Province (KJ2019A0267), Academic Funding Project for Top Talents in Colleges and Universities in Anhui Province (No. gxbjZD10), Nova Pew Plan of the Second Affiliated Hospital of Anhui Medical University (No. 2017KA01), Open Projects of Key Laboratory in Medical Physics and Technology of Anhui Province(LHJJ202001).

## Conflict of Interest

The authors declare that the research was conducted in the absence of any commercial or financial relationships that could be construed as a potential conflict of interest.

## Publisher’s Note

All claims expressed in this article are solely those of the authors and do not necessarily represent those of their affiliated organizations, or those of the publisher, the editors and the reviewers. Any product that may be evaluated in this article, or claim that may be made by its manufacturer, is not guaranteed or endorsed by the publisher.

## Supplementary Material

Supplementary Figure 1 Nineteen pyroptosis-related genes classify glioma patients into different clinicopathological features and OS in the CGGA dataset. **(A)** Cumulative distribution function of consensus clustering for k = 2 to 9. **(B)** The area under the CDF curve was relatively changed for k = 2 to 9. **(C)** The consensus clustering matrix showed that the 620 glioma patients from the CGGA dataset were grouped into two clusters (k = 2). **(D)** Survival analysis of glioma patients in cluster 1/2. **(E)** Heatmap of differential clinicopathologic features and nineteen pyroptosis-related gene expression levels between cluster 1 and cluster 2. ****p* < 0.001.

Supplementary Figure 2 Relationship among the cluster 1/2 subgroups, clinicopathological features of patients and the risk score in the CGGA dataset. **(A)** The heatmap shows the differential distribution of clinicopathological features and eleven pyroptosis-related gene expression levels in low and high-risk gliomas. **(B–I)** The WHO grade **(B)**, age **(C)**, fustat **(D)**, cluster 1/2 subgroups **(E)**, IDH status **(F)**, 1p/19q codel status **(G)**, chemotherapy status **(H)** and PRS type **(I)** stratify the CGGA dataset, and the distribution of risk scores is shown. **p* < 0.05, ***p* < 0.01 and ****p* < 0.001. **(J, K)** Univariate **(J)** and multivariate **(K)** analyses for the CGGA cohort including gender, age, grade and risk score.

Supplementary Figure 3 Differential expression, OS and DFS of the six pyroptosis-related genes. **(A–F)** Boxplots show the expression levels of HMOX1 **(A)**, IL18 **(B)**, IRAK4 **(C)**, RELB **(D)**, TIGAR **(E)** and TP53 **(F)** in normal brain tissue and GBM or low-grade glioma tissues. ****p* < 0.001 and *****p* < 0.0001. **(G–R)** Survival curves for OS and DFS of glioma patients with high expression and low expression of the following genes: HMOX1 **(G, M)**, IL18 **(H, N)**, IRAK4 **(I, O)**, RELB **(J, P)**, TIGAR **(K, Q)** and TP53 **(L, R)**.
