# Supplementary figures and images for: A Novel Defined Pyroptosis-Related Gene Signature for Predicting Prognosis and Treatment of Glioma
Source: Front Oncol. 2022 Mar 31;12:717926. doi: 10.3389/fonc.2022.717926 (PMC9008739; doi:10.3389/fonc.2022.717926)

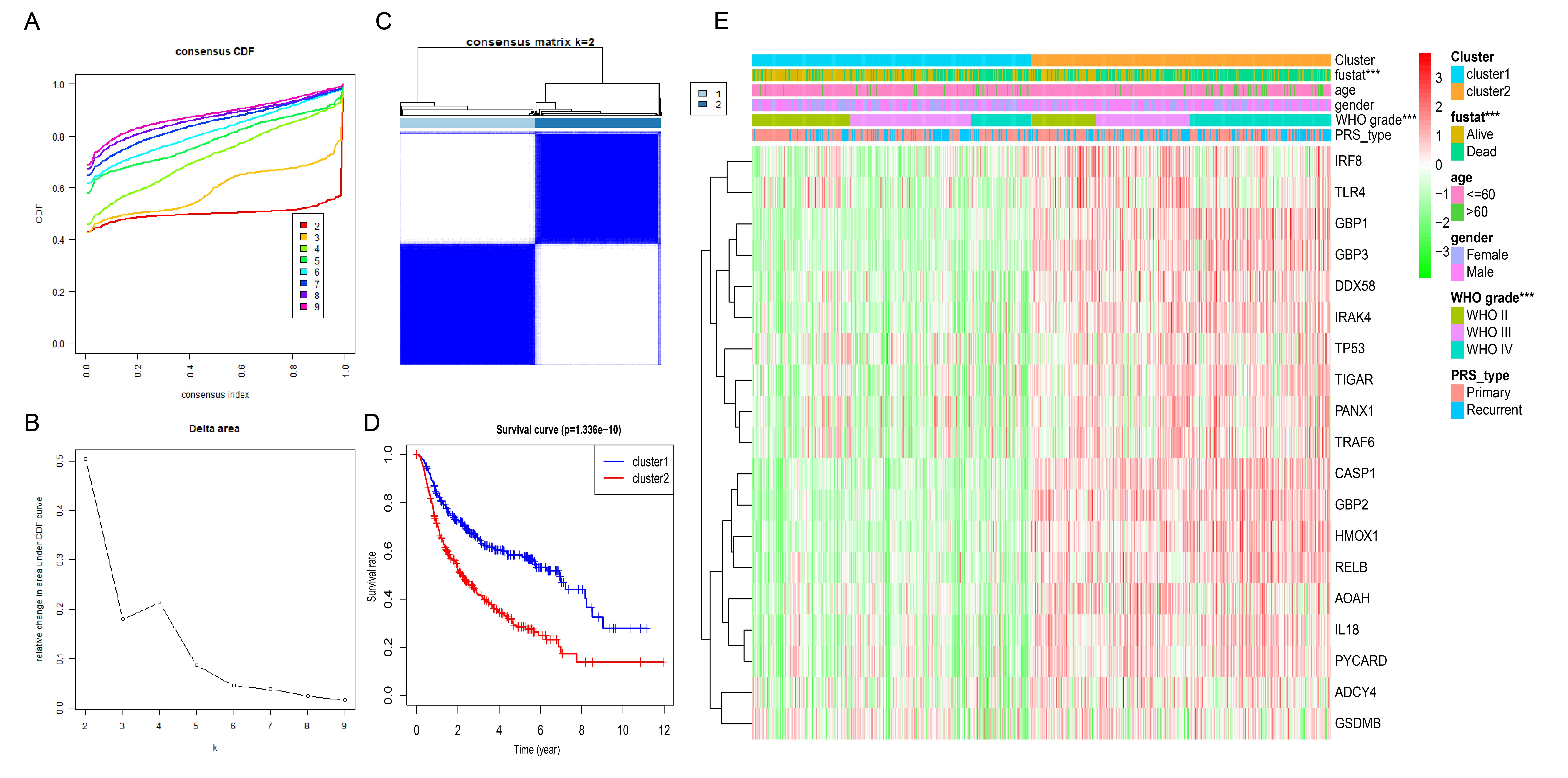

Supplement: Supplementary Figure 1 — Nineteen pyroptosis-related genes classify glioma patients into different clinicopathological features and OS in the CGGA dataset. (A) Cumulative distribution function of consensus clustering for k = 2 to 9. (B) The area under the CDF curve was relatively changed for k = 2 to 9. (C) The consensus clustering matrix showed that the 620 glioma patients from the CGGA dataset were grouped into two clusters (k = 2). (D) Survival analysis of glioma patients in cluster 1/2. (E) Heatmap of differential clinicopathologic features and nineteen pyroptosis-related gene expression levels between cluster 1 and cluster 2. ***p < 0.001. [file Image_1.jpeg]

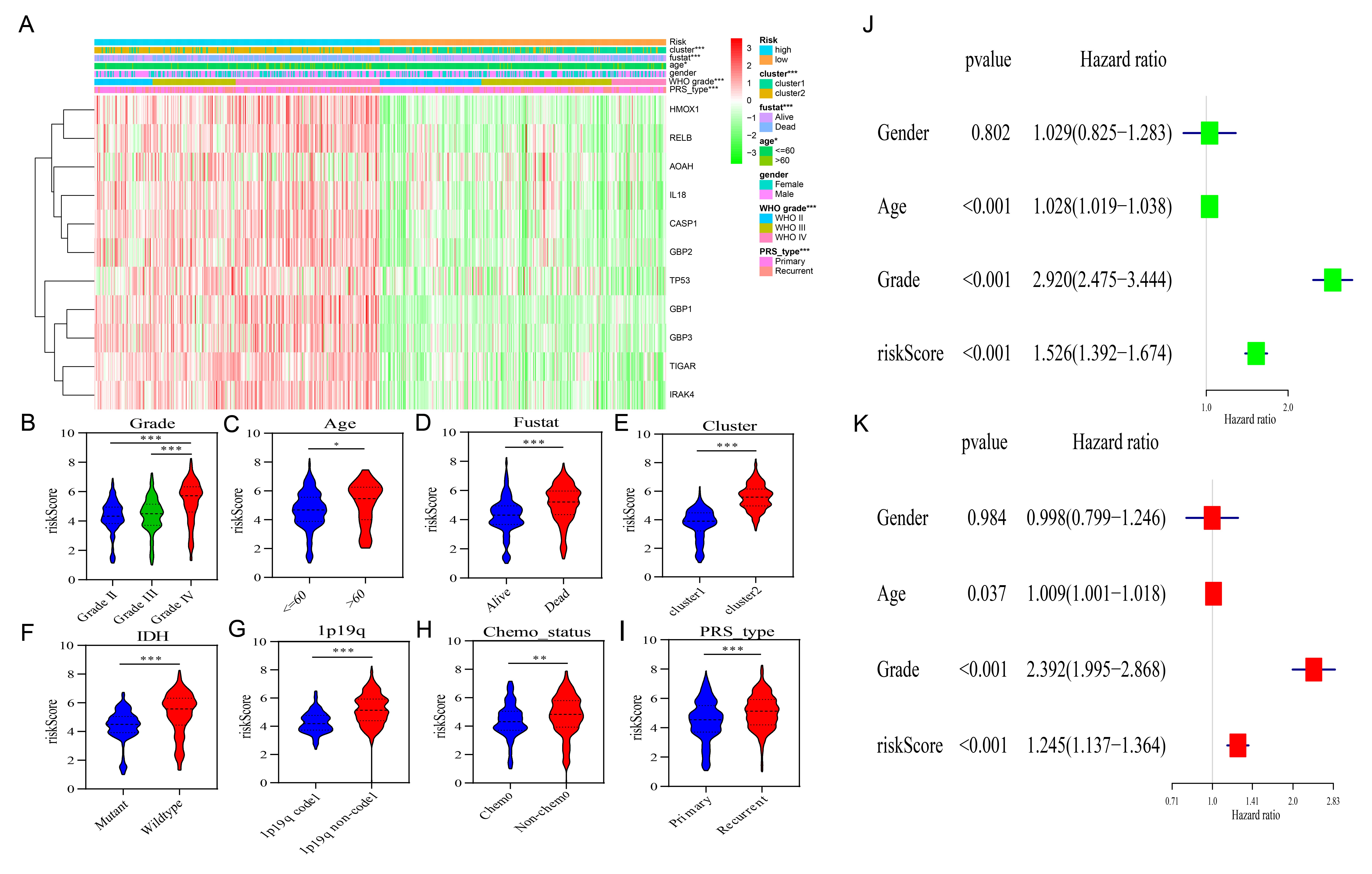

Supplement: Supplementary Figure 2 — Relationship among the cluster 1/2 subgroups, clinicopathological features of patients and the risk score in the CGGA dataset. (A) The heatmap shows the differential distribution of clinicopathological features and eleven pyroptosis-related gene expression levels in low and high-risk gliomas. (B–I) The WHO grade (B), age (C), fustat (D), cluster 1/2 subgroups (E), IDH status (F), 1p/19q codel status (G), chemotherapy status (H) and PRS type (I) stratify the CGGA dataset, and the distribution of risk scores is shown. *p < 0.05, **p < 0.01 and ***p < 0.001. (J, K) Univariate (J) and multivariate (K) analyses for the CGGA cohort including gender, age, grade and risk score. [file Image_2.jpeg]

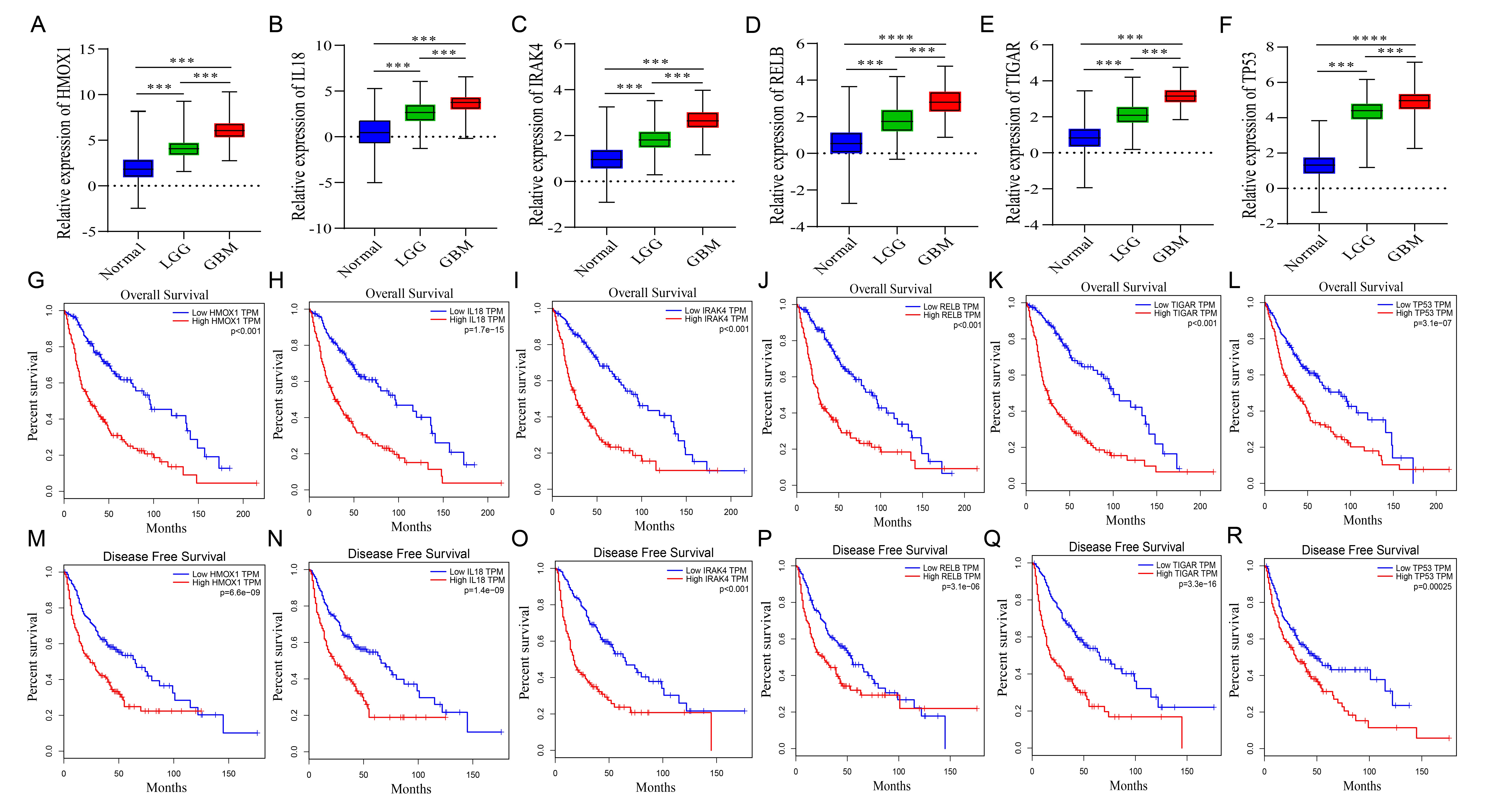

Supplement: Supplementary Figure 3 — Differential expression, OS and DFS of the six pyroptosis-related genes. (A–F) Boxplots show the expression levels of HMOX1 (A), IL18 (B), IRAK4 (C), RELB (D), TIGAR (E) and TP53 (F) in normal brain tissue and GBM or low-grade glioma tissues. ***p < 0.001 and ****p < 0.0001. (G–R) Survival curves for OS and DFS of glioma patients with high expression and low expression of the following genes: HMOX1 (G, M), IL18 (H, N), IRAK4 (I, O), RELB (J, P), TIGAR (K, Q) and TP53 (L, R). [file Image_3.jpeg]
